# Corrigendum: Chimera-like States in Modular Neural Networks

**DOI:** 10.1038/srep22314

**Published:** 2016-03-04

**Authors:** Johanne Hizanidis, Nikos E. Kouvaris, Gorka Zamora-López, Albert Díaz-Guilera, Chris G. Antonopoulos

Scientific Reports 6: Article number: 19845; 10.1038/srep19845 published online: 01222016; updated: 03042016.

The original version of this Article contained an error in the spelling of the author Gorka Zamora-López, which was incorrectly given as Zamora-López Gorka. This has now been corrected in the PDF and HTML versions of the Article.

