# Supplementary material for: Chimera-like States in Modular Neural Networks
Source: Sci Rep. 2016 Jan 22;6:19845. doi: 10.1038/srep19845 (PMC4726386; doi:10.1038/srep19845)
Supplement: Supplementary Information [file srep19845-s4.pdf]

Chimera-like states in modular neural networks
# – Supplementary Information –

Johanne Hizanidis,[1,2] Nikos E. Kouvaris,[3] Gorka Zamora-López,[4,5] Albert Díaz-Guilera,[3] and Chris G. Antonopoulos[6]

[1]*Crete Center for Quantum Complexity and Nanotechnology, Physics Department, University of Crete, 71003 Heraklion, Greece*
[2]*National Center for Scientific Research "Demokritos", 15310 Athens, Greece*
[3]*Department of Physics, University of Barcelona, Martí i Franquès 1,E-08028, Barcelona, Spain*
[4]*Center for Brain and Cognition, Universitat Pompeu Fabra, Barcelona, Spain*
[5]*Department of Information and Communication Technologies, Universitat Pompeu Fabra, Barcelona, Spain*
[6]*Department of Mathematical Sciences, University of Essex, Wivenhoe Park, CO4 3SQ Colchester, UK*

In this Supplementary Information, we provide the following:

I. Details of the supplementary movies. The movies are available online separately.

II. Analysis of the modular network with communities detected with the Louvain method.

III Analysis for the modular network with random small-world communities.

IV. Analysis for the modular network with random Erdős-Rényi communities.

I. Supplementary movies

Details of supplementary movies which correspond to Figures 3((a)-(c)) of the main text are provided here. The movies are available online separately. They illustrate the nonlinear evolution of synchronous (Figure 3(a)), metastable (Figure 3(b)) and chimera-like (Figure 3(c)) dynamics.

Supplementary Movie S1. It shows the time evolution of the Hindmarsh-Rose model, as it is described by Eqs. (1). The strength of electrical and chemical coupling is $g_{el} = 1.7$ and $g_{ch} = 0.015$, respectively that correspond to point **A**, marked in the parameter space in Figure 2. The time growth is illustrated by a gray horizontal line which traverses the plots of spacetime and time-series and moves vertically towards increasing time. The dynamical behavior shown in this movie is **synchronous** (see also Figure 3(a)).

Supplementary Movie S2. It shows the time evolution of the Hindmarsh-Rose model, as it is described by Eqs. (1). The strength of electrical and chemical coupling is $g_{el} = 0.7$ and $g_{ch} = 0.18$, respectively that correspond to point **B**, marked in the parameter space in Figure 2. The time growth is illustrated by a gray horizontal line which traverses the plots of spacetime and time-series and moves vertically towards increasing time. The dynamical behavior shown in this movie is **desynchronous** (see also Figure 3(b)).

Supplementary Movie S3. It shows the time evolution of the Hindmarsh-Rose model, as it is described by Eqs. (1). The strength of electrical and chemical coupling is $g_{el} = 0.5$ and $g_{ch} = 0.015$, respectively that correspond to point **C**, marked in the parameter space in Figure 2. The time growth is illustrated by a gray horizontal line which traverses the plots of spacetime and time-series and moves vertically towards increasing time. The dynamical behavior shown in this movie is **chimera-like**, the communities 1 (brown), 3 (red) and 5 (purple) are desynchronized, whereas the rest are synchronized (see also Figure 3(c)).

II. Analysis of the modular network with communities detected with the Louvain method

Here, we proceed to the same analysis presented in the main text by using a modular network with 277 neurons grouped into six communities. The communities are detected by employing the Louvain method [1]; they are shown in Figure S1. As is discussed in the text, neurons of the same community are assumed to be connected with electrical coupling, while accross communities with chemical coupling.

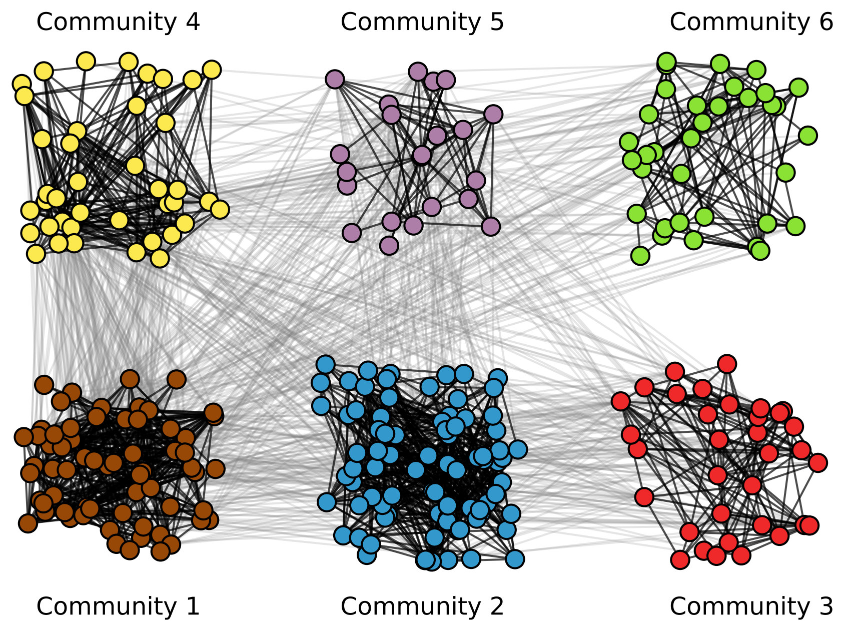

Figure S1: **Modular organization of the *C.elegans* based neural network.** Employing the Louvain method [1], six communities have been detected in the neural network of *C.elegans*. Different colors are assigned to neurons of different communities, 1: brown (64 neurons), 2: blue (82 neurons), 3: red (34 neurons), 4: yellow (40 neurons), 5: purple (22 neurons), 6: green (35 neurons); Black links indicate electrical coupling between neurons within the same community and gray represent chemical couplings between neurons across different communities.

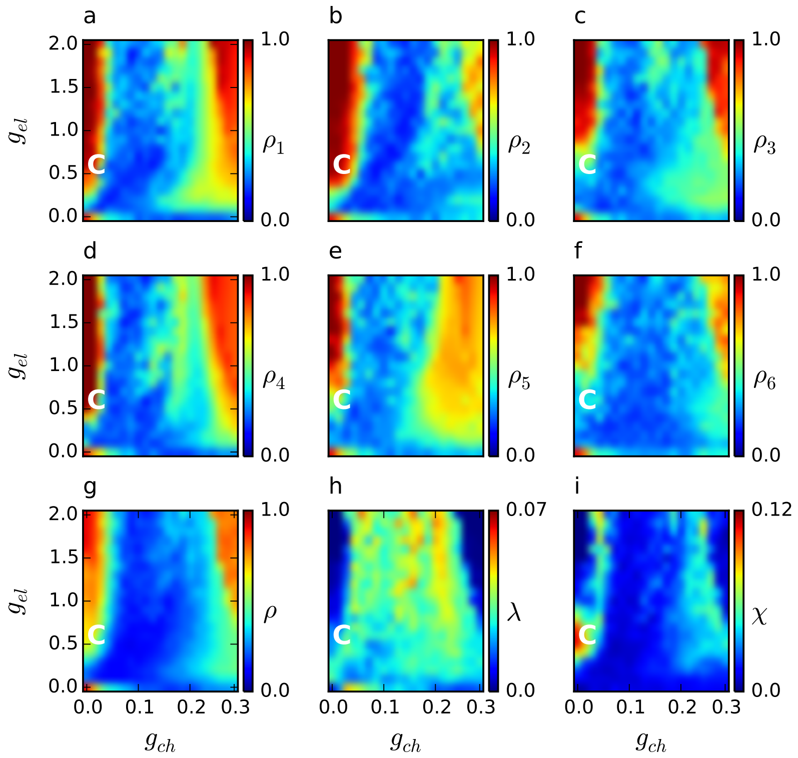

Figure S2: **Parameter spaces.** Parameter spaces in the (g_{ch}, g_{el}) plain show the order parameter (synchronization) of each community ρ_i, $i = 1, \ldots, 6$ ((a)-(f)), the entire network ρ (g), the metastability index λ (h) and the chimera-like index χ (i). All indices are calculated for the modular network of FIG. S1.

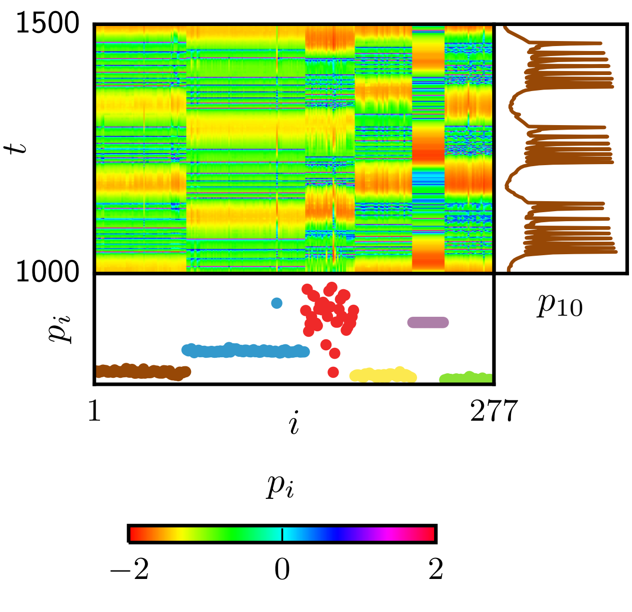

Figure S3: **Space-time plots.** A chimera-like state is shown for $g_{el} = 0.5$ and $g_{ch} = 0.03$, values which correspond to point **C** in Fig. S2.

III. Analysis for modular networks with small-world communities

Here, we proceed to the same analysis presented in the main text by using a modular network with 277 neurons grouped into six communities. Each community is a subnetwork created with the configuration model by randomly assigning links to match the degrees sequences (*i.e.* the number of electrical synapses of the i-th neuron) as they were detected in the *C.elegans* neural network using the walktrap algorithm. We eliminate parallel links and self loops. Then, we connect neurons across communities with a random network, whose links represent the chemical synapses. The number of these synapses is the same as in the network used in the main text.

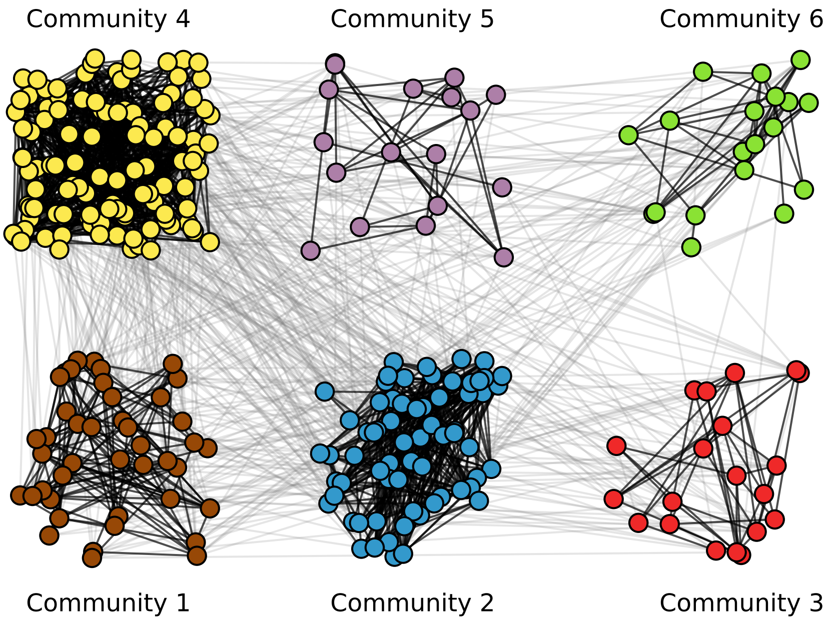

Figure S4: **Modular network with small-word communities.** A modular network with six interconnected communities is shown. We assign a number and color to each community (1: brown, 2: blue, 3: red, 4: yellow, 5: purple, 6: green). Black links indicate electrical couplings between neurons within the same community, whereas gray links represent chemical couplings between neurons across different communities.

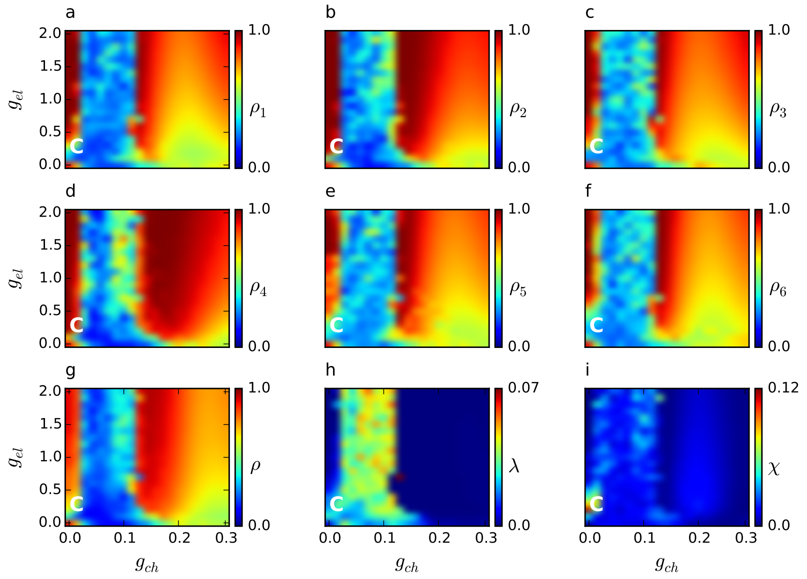

Figure S5: **Parameter spaces.** Parameter spaces in the (g_{ch}, g_{el}) plain show the order parameter (synchronization) of each community ρ_i, $i = 1, \ldots, 6$ ((a)-(f)), the entire network ρ (g), the metastability index λ (h) and the chimera-like index χ (i). All indices are calculated for the modular network of FIG. S4

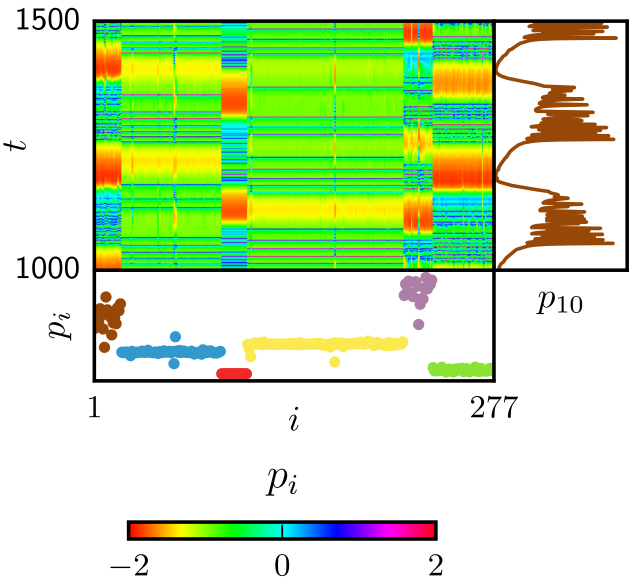

Figure S6: **Space-time plots.** A chimera-like state is shown for $g_{el} = 0.5$ and $g_{ch} = 0.04$, values which correspond to point **C** in Fig. S5.

IV. Analysis for modular networks with random communities

Here, we proceed to the same analysis presented in the main text by using a modular network with 277 neurons grouped into six Erdős-Rényi communities. Each of the six communities has the same number of neurons with the communities detected in the *C.elegans* neural network using the walktrap algorithm. Moreover, we use the same number of electrical synapses, and we randomly assign them to a pair of neurons with equal probability. By doing this, each community is an Erdős-Rényi subnetwork. Chemical synapses used here are the same as in the modular networks with small-world communities, discussed previously in the supplementary material.

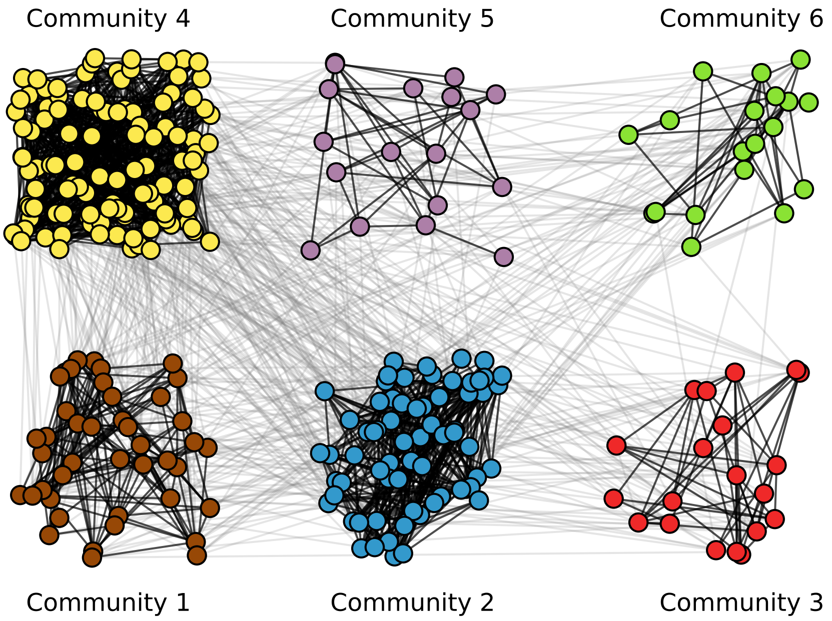

Figure S7: **Modular network with random communities.** A modular network with six interconnected Erdős-Rényi communities is shown. We assign a number and color to each community (1: brown, 2: blue, 3: red, 4: yellow, 5: purple, 6: green). Black links indicate electrical coupling between neurons within the same community and gray represent chemical couplings between neurons across different communities.

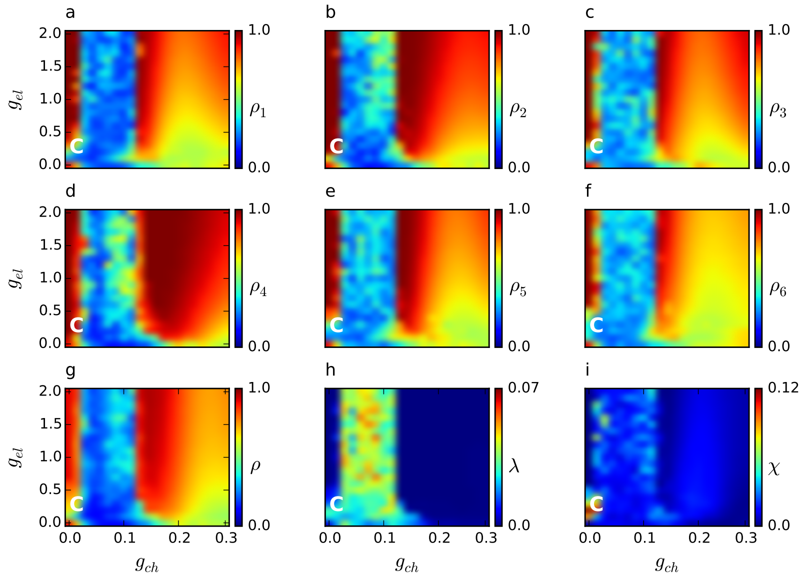

Figure S8: **Parameter spaces.** Parameter spaces in the (g_{ch}, g_{el}) plain show the order parameter (synchronization) of each community ρ_i, $i = 1, \ldots, 6$ ((a)-(f)), the entire network ρ (g), the metastability index λ (h) and the chimera-like index χ (i). All indices are calculated for the modular network of FIG. S7.

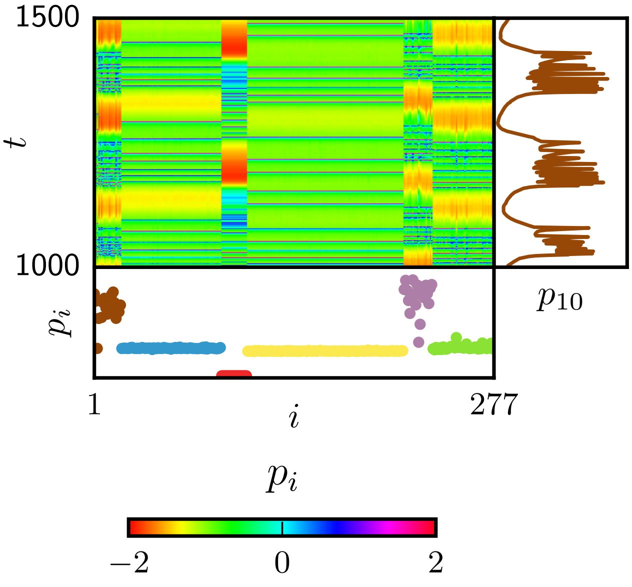

Figure S9: **Space-time plots.** A chimera-like state is shown for $g_{el} = 0.5$ and $g_{ch} = 0.03$, values which correspond to point **C** in Fig. S8.

[1] Blondel, V. D., Guillaume, J. L., Lambiotte, R. & Lefebvre, E. "Fast unfolding of communities in large networks.", Journal of Statistical Mechanics: Theory and Experiment 2008, P10008 (2008).
